# Temporal dysfunction in traumatic brain injury patients: primary or secondary impairment?

**DOI:** 10.3389/fnhum.2014.00269

**Published:** 2014-04-30

**Authors:** Giovanna Mioni, Simon Grondin, Franca Stablum

**Affiliations:** ^1^École de Psychologie, Université LavalQuébec, QC, Canada; ^2^Department of General Psychology, University of PadovaPadova, Italy

**Keywords:** traumatic brain injury, time perception, time reproduction, time production, time discrimination, executive functions

## Abstract

Adequate temporal abilities are required for most daily activities. Traumatic brain injury (TBI) patients often present with cognitive dysfunctions, but few studies have investigated temporal impairments associated with TBI. The aim of the present work is to review the existing literature on temporal abilities in TBI patients. Particular attention is given to the involvement of higher cognitive processes in temporal processing in order to determine if any temporal dysfunction observed in TBI patients is due to the disruption of an internal clock or to the dysfunction of general cognitive processes. The results showed that temporal dysfunctions in TBI patients are related to the deficits in cognitive functions involved in temporal processing rather than to a specific impairment of the internal clock. In fact, temporal dysfunctions are observed when the length of temporal intervals exceeds the working memory span or when the temporal tasks require high cognitive functions to be performed. The consistent higher temporal variability observed in TBI patients is a sign of impaired frontally mediated cognitive functions involved in time perception.

Adequate temporal abilities are important to perform most of everyday activities and understanding how human perceive time is always an engaging question. Good temporal skills are essential for normal social functioning, such as crossing a busy street, preparing a meal or organizing the daily activities. Indeed, humans have to process time across a wide range of intervals, from milliseconds up to the hour range (Fraisse, 1984; Pöppel, 2004; Buhusi and Meck, 2005; Grondin, 2010).

One of the most influential models of time processing, the Scalar Expectancy Theory (SET; Gibbon et al., 1984) assumes that temporal judgments are based on three processing stages: the clock, memory, and decision stages. According to the SET model, the first stage consists of a pacemaker emitting pulses; these pulses pass through a switch and are stored into an accumulator. The content of the accumulator provides the raw material for estimating time (clock stage). The outcome from the accumulator is stored in the working memory system for comparison with the content in the reference memory, which contains a long-term memory representation of the number of pulses accumulated on past trials (memory stage). Finally, a decision process compares the current duration values with those in working and reference memory to decide on the adequate temporal response (decision stage).

Errors in temporal processing may depend on different factors and occur at each stage of the SET model. Variations in the rate of pulses' emission by the pacemaker are often reported to be an important cause of temporal errors. These variations have several causes like changes in body temperature (Hancock, 1993; Aschoff, 1998), experiencing emotions (Angrilli et al., 1997; Droit-Volet et al., 2013; Grondin et al., in press) and using pharmacological substances (Meck, 1996; Rammsayer, 2008). The switch is the part of the clock process that is directly associated with the mechanisms of attention. When the switch is closed, the pulses that are emitted by the pacemaker are accumulated in the counter. Indeed, it is the amount of attention paid to time that determines the accumulation of pulses in the counter. The demonstration of the role of attention in temporal processing is often based on the dual-task paradigm, in which attention has to be divided between temporal and non-temporal tasks. Results showed that when more attention is dedicated to time, more pulses are accumulated in the counter and less temporal errors are produced (Zakay and Block, 1996, 2004; Block and Zakay, 2006). When subjects are asked to estimate time and execute other cognitive tasks, the accuracy of time estimation is reduced because time estimation shares attentional resources with the non-temporal tasks and the amount of the shared resources depends on the nature of the second task (Brown, 1997). Finally, a part of the variance in the processing of time depends on memory and decisional processes (Penney et al., 2000; Pouthas and Perbal, 2004; Wittmann and Paulus, 2008). In fact, the quality of the interval's representation in reference memory is a source of variability in temporal processing (Pouthas and Perbal, 2004; Grondin, 2005). When the content of the accumulator is transferred to working memory for the comparison with the content stored in reference memory, the temporal representation retrieved from the reference memory might have been modified according to the characteristics of the memory system (Harrington and Haaland, 1999; Penney et al., 2000; Ogden et al., 2008).

## Different temporal ranges and different methods for investigating time perception

For investigating time perception, two factors are critical, namely the temporal range (Grondin, 2001, 2012) and the method employed (Zakay, 1990, 1993; Grondin, 2008; Tobin et al., 2010). Regarding the temporal range, very brief intervals have received special attention because they are directly involved in motor coordination and in the processing of speech and music (Pöppel, 2004; Grondin, 2010). There are reasons to believe that distinct temporal processes are involved with intervals above vs. below 1 s (Penney and Vaitilingam, 2008; Rammsayer, 2008). While the basal ganglia and the cerebellum are involved in the processing of both the short and the long intervals, the contribution of the prefrontal regions seems limited to the processing of long intervals (Meck, 2005; Rubia, 2006). Indeed, the cerebellum and basal ganglia would be related to the internal clock mechanism, cognitive functions necessary to complete a temporal task being assumed by the prefrontal areas.

Traditionally, authors distinguish four methods for investigating time perception: time production, verbal estimation, time reproduction and time discrimination (Allan, 1979; Block, 1989; Zakay, 1993; Mangels and Ivry, 2000; Gil and Droit-Volet, 2011a). There are many other methods described in the timing and time perception literature (Grondin, 2008, 2010), but for the sake of the present review, it is relevant to focus on classical ones. Time production and verbal estimation tasks may be considered the two sides of the same coin and reflect the same underlying temporal processes and mechanisms (Allan, 1979; Block, 1990). In time production tasks a participant has to produce an interval equal to an interval previously reported (i.e., “Produce 2 s”). In the verbal estimation tasks, after experiencing target duration, a participant has to translate this subjective duration into clock units. Time production and verbal estimation are appropriate ways for investigating individual differences related to the internal clock (its speed rate or the variables influencing it). Because humans have a tendency to round off the time estimates with chronometric units, verbal estimations produce more variability and is less accurate than time production method. In time reproduction tasks, after first experiencing target duration, a participant is asked to delimit a time period, usually with finger taps, equivalent the target duration (Mioni et al., 2014). Compared to time production or verbal estimation tasks, a time reproduction task is less used to investigate individual differences at the internal clock level. In fact, the speed rate of the internal clock is the same when experiencing the target duration and when reproducing it. Finally, in time discrimination tasks, a participant has to compare the relative duration of two successive intervals (standard—comparison) by indicating which one was longer or shorter. Note that a time-order error (TOE) is often observed when performing a time discrimination task with the presentation of two successive stimuli. The TOE is defined as positive if the first stimulus is over-estimated or as negative if the first stimulus is under-estimated relative to the second stimulus (Hellström, 1985; Eisler et al., 2008). Just like with the time reproduction method, any clock rate variation would not be detected with a time discrimination task because the processing of both the standard and the comparison intervals would be affected (Zakay, 1990; Rammsayer, 2001; Mioni et al., 2013a).

Researchers are using the entire repertory of methods but in most cases they give no explanation for the selection of a specific one. It is obvious that each method activates different time-related processes and presents some specific perceptual errors. For example, participants tested with the verbal estimation methods are prone to respond to the estimated duration in round number and produced a great amount of variability compared to the other methods (Zakay, 1990; Grondin, 2010). Time reproduction is considered to be more accurate and reliable than time production and verbal estimation; however, it is less useful for investigating variations in the pacemaker rate. Block (1989) noted that time production and verbal estimation show more inter-subject variability than time reproduction or time discrimination, but can be successfully used in studies where the rate of the internal pacemaker is manipulated. Others have pointed out that time discrimination is the purest measure of time perception because briefer intervals can be used, limiting the involvement of additional cognitive processes caused by the processing of long temporal intervals (Rubia et al., 1999; Block and Zakay, 2006; Mioni et al., 2013b). However, the time discrimination task is prone to TOE (Eisler et al., 2008).

Taken into consideration that each method activates different time-related processes, one way to select the appropriate method is to take the temporal interval under investigation into account (Gil and Droit-Volet, 2011a). Time discrimination tasks are often chosen for very brief intervals (from 50 ms up to a few seconds) while verbal estimation, time production, and time reproduction tasks are often used with longer intervals (Grondin, 2008, 2010).

Data collected from time reproduction, time production and verbal estimation tasks may be scored in term of absolute score, relative error and/or coefficient of variation. Briefly, the absolute score reflects the errors' magnitude, regardless its direction (Brown, 1985; see also Glicksohn and Hadad, 2012). The relative error reflects the direction of the timing error. It is measured by dividing the estimated duration (*E*_*d*_) of the participant by the target duration (*T*_*d*_) (RATIO = *E*_*d*_/*T*_*d*_). A score of 1 means that the estimation is perfect; a score above 1 reflects an overestimation; and a score below 1 means that the interval was underestimated. Finally, the coefficient of variance (CV) is an index of timing variability over a series of trials. The CV is the variability (for instance, one standard deviation) divided by the mean judgments. In the case of time discrimination tasks, performance is analyzed in terms of sensitivity and perceived duration (Grondin, 2008, 2010). Depending on the exact method used for discriminating intervals, different dependent variables can be used. For instance, for sensitivity, it could be the proportion of correct responses, *d*′, difference threshold or a coefficient of variation (difference threshold divided by the bisection point); and, for perceived duration, it could be the proportion of “long” responses, *c*, or a bisection point on a psychometric function.

## Cerebral bases of temporal processing

Different brain areas have been identified to play a critical role in temporal processing. By identifying the brain areas and networks responsible for governing temporal processing, researchers can now study the reasons of temporal impairment. Studies have shown that patients with focal lesions to frontal brain regions (both right and left frontal areas) are impaired in their ability to estimate temporal intervals (Nichelli et al., 1995; Rubia et al., 1997; Harrington et al., 1998; Mangels et al., 1998; Casini and Ivry, 1999). In particular, the integrity of the right dorso-lateral prefrontal cortex and right inferior parietal lobe has been shown to be necessary for the discrimination and estimation of intervals of several seconds (Rubia et al., 1997; Harrington et al., 1998; Mangels et al., 1998; Kagerer et al., 2002). The importance of the cerebellum in timing processes is also well-established. Patients with cerebellar lesions showed poor performances on both motor tapping and time estimation tasks, both in the range of hundreds of milliseconds and of a few seconds (Ivry and Keele, 1989; Ivry and Diener, 1991; Harrington et al., 2004; Gooch et al., 2010). The role of the basal ganglia in time estimation and motor timing functions is confirmed by studies with Parkinson's disease patients showing deficits in motor timing and time perception that can be improved with dopaminergic treatments (Jones et al., 2008; Merchant et al., 2008). Finally, the parietal cortex is also emerging as an important locus of multimodal integration of time, space and numbers and the right inferior parietal cortex seems to be necessary for rapid discrimination of temporal intervals (Walsh, 2003a,b; Alexander et al., 2005; Bueti and Walsh, 2009; Hayashi et al., 2013).

However, most of the brain areas and networks involved in temporal processing are also involved in other cognitive functions (Kane and Engle, 2002; Busch et al., 2005; Aharon Peretz and Tomer, 2007). While frontally mediated cognitive processes (i.e., attention, working memory, executive functions, etc.) play an important role in temporal processing (Rao et al., 2001; Perbal et al., 2002; Baudouin et al., 2006a,b; Mioni et al., 2013a,b), frontally mediated cognitive deficits are well-documented in traumatic brain injury (TBI) patients (Azouvi, 2000; Leclercq et al., 2000; Boelen et al., 2009; Stuss, 2011).

## Time perception in traumatic brain injury patients

Temporal impairments in patients with TBI are expected considering the disruption of cognitive functions involved in temporal processing. However, what is less clear is whether TBI patients present a “pure” temporal impairment due to disruption of some brain areas and of the network specifically involved in temporal processing, or present a temporal dysfunction mainly because of an impairment of the cognitive functions involved in temporal processing.

### Main characteristics of TBI patients

TBI presents unique problems to its survivors, their relatives and others involved in their rehabilitation. It occurs predominantly in young adults, most commonly males. Neuropathological evidences suggest a marked heterogeneity of injuries across individuals and the delineation of the precise nature and extent of an injury in an individual might be very difficult. However, it is apparent that diffuse axonal injury is common, and that damage occurs most frequently in the frontal and temporal lobes. TBI usually results in immediate loss or impairment of consciousness, followed by a period of confusion. Following the return of orientation, TBI patients exhibit sensorimotor, cognitive and behavioral sequels, which vary widely in their severity. In the majority of cases, it is the cognitive changes which are most disruptive and disabling in the long term. These may include deficits of attention, speed of processing, memory, planning and problem solving, and lack of self-awareness (Ponsford et al., 1995; Lezak, 2004).

Although investigating time perception in TBI patients is of particular interest from both a clinical and experimental point of view, there is not much empirical work on the temporal dysfunctions of these patients. Indeed, TBI patients often report such dysfunctions. Considering that an impaired sense of time could affect the daily adaptive functioning of patients recovering from TBI, understanding fully the causes of the temporal impairments observed in TBI patients is crucial. In addition to contribute to the understanding of the brain areas and networks involved in temporal processing, studying temporal dysfunctions in TBI patients should conduct to the elaboration of appropriate rehabilitation programs.

### Methodological issues

A computer-based search involving PsycInfo, PubMed and Web of Science was conducted using the terms: TBI, closed head injury, temporal perception, time estimation, time reproduction, time production, time discrimination, duration reproduction and duration production. In addition, reference lists from published reviews, books, and chapters were checked to identify studies that may not have been found when searching on databases. The research was conducted independently by the first author and by the library assistance at Padova University, and covered a period from 1950 to February 2014. These search methods resulted in a combined total of 88 published articles. Only studies involving specifically TBI patients and matched controls that performed temporal tasks (i.e., time reproduction, time production, verbal estimation, and time discrimination tasks) were included in the present review. Out of the 88 papers identified, 27 articles were found in more than one computer-based source. Out of the 61 different articles, were excluded from the review five articles reporting animal data, two dissertation abstracts, 18 papers reporting data with other patients (cerebellar patients, autistic patients, etc.), and 27 articles in which it was not a timing or time perception task that was used, but tasks related for instance to processing speed deficits, time recover after TBI, or temporal context memory. Finally, two articles were also excluded because they did not report new data, but data that have been published earlier in other articles.

In the end, in spite of the importance of adequate temporal abilities in everyday activities, only seven studies investigating time perception following TBI were identified and included in the present work (Meyers and Levin, 1992; Perbal et al., 2003; Schmitter-Edgecombe and Rueda, 2008; Anderson and Schmitter-Edgecombe, 2011; Mioni et al., 2012, 2013a,b). Table 1 provides a summary of the findings reported in these articles.

**Table 1:** **Summary table of studies that have investigated time perception in TBI patients**.

**References**	**Patients**	**Clinical characteristics**	**Tasks**	**Condition**	**Durations**	**Neuropsychological tasks^a^**	**Effect size**
Meyers and Levin, 1992	Disoriented TBI = 10	18 severe, 5 moderate, 11 mild; PTA = 16; TPI = 37 days	Time reproduction	Simple	5, 10, and 15 s	NA	5 s *d* = 1.01^b^^,^^c^
	Oriented TBI = 24		Verbal estimation				10 s *d* = 0.46^b^^,^^c^
	Controls = 12						15 s *d* = 0.52^b^^,^^c^
Perbal et al., 2003	TBI = 15	GCS = 6.4; PTA = 88; TPI = 11 months	Time reproduction	Control counting	5, 14, and 38 s	Short-term memory = forward digit and spatial span, Corkin-free interval; Working memory = backward digit and spatial span, Corkin-concurrent interval; Episodic memory = Grober and Buschke task	NA
	Controls = 15		Time production	Concurrent reading			
Schmitter-Edgecombe and Rueda, 2008	TBI = 27	14 severe and 13 moderate; PTA = 24.11; TPI = 41.93 days	Verbal estimation	Concurrent reading	10, 25, 45, and 60 s	*Attention/speeded processing* = Symbol Digit Modalities Test, Trail Making Test—A; *Verbal memory* = Rey Verbal Learning Test; *Visual memory* = 7/24 Spatial Recall Test; *Executive functions* = Trial Making Test—B, Letter Number Sequencing subtest from the WAIS-III, Controlled Oral Word Association Test	10 s *d* = 0.29^b^
	Controls = 27						25 s *d* = 0.51^b^
							45 s *d* = 0.91^b^
							60 s *d* = 0.96^b^
Anderson and Schmitter-Edgecombe, 2011	TBI = 15	8 severe and 7 moderate; PTA = 23.27; TPI = 432.13 days	Verbal estimation	Concurrent reading	10, 25, 45, and 60 s	*Attention/Speeded processing* = Symbol Digit Modalities Test, Trial Making Test-A; *Episodic memory* = Rey Auditory; Verbal Learning Test, 7/24 Spatial Recall Test; *Executive functions* = Letter Number; Sequencing subtest from the WAIS-III, Controlled Oral Word Association Test	η^2^ = 0.16^d^
	Controls = 15						
Mioni et al., 2012	TBI = 18	18 severe; GCS = 6.77; PTA = 11.78; TPI = 24.50	Time reproduction	Concurrent reading	4, 9, and 14 s	*Attention* = Stroop; *Working memory* = N-back	4 s *d* = 0.05^b^
	Controls = 18						9 s *d* = 0.05^b^
							14 s *d* = 0.04^b^
Mioni et al., 2013a	TBI = 27	27 severe; GCS = 7; PTA = 62.81; TPI = 30.15	Time discrimination	Simple	Standard duration 500 and 1300 ms	*Attention* = Stroop; *Working memory* = N-back	500 ms *d* = 1.5^e^
	Controls = 27						1300 ms *d* = 0.75^e^
Mioni et al., 2013b	TBI = 15	15 severe; GCS = 6.3; TPI = 31.40	Time reproduction	Simple	500, 1000, and 1500 ms	*Attention* = Divided attention, Go-Nogo; *Working memory* = N-back, Digit span backward; *Executive functions* = Verbal fluency, Wisconsin Cards Sorting Test	Time reproduction^b^: 500 ms *d* = 0.47; 1000 ms *d* = 0.17; 1500 *d* = 0.23 Time production^b^: 500 ms *d* = 0.05; 1000 ms *d* = 0.81; 1500 *d* = 0.06 Time discrimination^e^: 500 ms *d* = 0.88; 1000 ms *d* = 0.72; 1500 *d* = 0.62
	Controls = 15		Time production				
			Time discrimination				

GCS, Glasgow Coma Scale (Teasdale and Jennett, 1974; <8 = severe TBI); TPI, Time Post Injury; PTA, Post Traumatic Amnesia (Mean days); NA, not available; Simple, temporal task alone; Control counting, count aloud for the stimulus duration; Concurrent reading, temporal task + non-temporal task.

a References to neuropsychological tasks are not reported because the authors referred to different versions of the tasks; please refer to the specific articles for the appropriate references.

b Analyses were conducted on RATIO.

c Analyses were conducted between TBI patients (oriented and disoriented TBI together) and controls.

d Analyses were conducted on RATIO as reported in the original paper.

e Analyses were conducted on proportion of correct responses.

### Approaching the literature from a method perspective

Among the study selected, 4 included the performances on a time reproduction task (Meyers and Levin, 1992; Perbal et al., 2003; Mioni et al., 2012, 2013b), 3 on a verbal estimation task (Meyers and Levin, 1992; Schmitter-Edgecombe and Rueda, 2008; Anderson and Schmitter-Edgecombe, 2011), 2 on a time production task (Perbal et al., 2003; Mioni et al., 2013b), and 2 on time discrimination task (Mioni et al., 2013a,b).

The studies conducted with the time reproduction task showed that TBI patients were as accurate as controls (RATIO) and showed higher variability (CV), indicating dysfunction in maintaining a stable representation of the temporal intervals. In the study conducted by Perbal et al. (2003), participants were also asked to perform a secondary task (non-temporal task) together with the time reproduction task to investigate the effect of reduced attentional resources on time perception. Similar RATIO was observed in TBI patients and controls in both simple (time reproduction only) and concurrent (time reproduction + non-temporal task) conditions. Both TBI patients and controls under-reproduced temporal intervals, in particular when the secondary non-temporal task was performed together with the time reproduction task. When the CVs were taken into consideration, TBI patients were more variable than controls when the secondary task was included.

The studies conducted with a time production task confirmed the results obtained with the time reproduction task. TBI patients were as accurate as controls (RATIO) but showed higher temporal variability (CV) (Perbal et al., 2003; Mioni et al., 2013b). Regarding the impact of a concurrent non-temporal task, no effect was found (time production only vs. time production + non-temporal task) and this finding applies to both groups. TBIs and controls showed the same performances (RATIO and CV) in both simple and concurrent conditions (Perbal et al., 2003).

Three studies were conducted with a verbal estimation task but performance was analyzed only in two of them. Indeed, in Meyers and Levin's (1992) study, performance at verbal estimation task was not analyzed due to the extreme variability noted in the TBI sample. Schmitter-Edgecombe and Rueda (2008), as well as Anderson and Schmitter-Edgecombe (2011), reported lower accuracy (absolute score), higher under-estimation (RATIO) and more variability (CV) in TBI patients than controls.

Finally, two studies were conducted with a time discrimination task. TBI patients were less accurate (proportion of correct responses) and more variable (CV) than controls (Mioni et al., 2013a,b). Moreover, Mioni et al. (2013a) examined the TOE in the time discrimination task. TBI showed a greater TOE than controls, indicating a bias in responding “short” when the standard was 500 ms (positive TOE) and responding “long” when the standard was 1300 ms (negative TOE). It is worth mentioning that a TOE is always observed in a time discrimination task (Hellström, 1985), but that the magnitude is greater in TBI patients.

In brief, TBI patients and controls have similar performances (absolute score or RATIO) when time reproduction and time production tasks are employed. However, TBI patients performed less accurately than controls when verbal estimation and time discrimination tasks were used. Moreover, in all studies, variability is higher with TBI patients than with controls.

### Approaching the literature from a temporal range perspective

A review as a function of the length of the intervals under investigation first reveals that most studies (5 out of 7) are concerned with long intervals (between 4 and 60 s). Lower performances are observed only when temporal intervals are longer than 45 s, probably because the temporal intervals exceed the working memory span (Mimura et al., 2000). In the range between 4 and 38 s, TBI patients seem to be as accurate as controls in terms of absolute score and RATIO. Only two studies have investigated temporal abilities in TBI patients with short durations (in the range of milliseconds to a few seconds), which might be particularly interesting considering that some of everyday activities are executed within this time range (Block, 1990; Block et al., 1998; Pöppel, 2004). Moreover, by employing short durations, there is a reduced load of higher cognitive processes because the processing of temporal intervals below 1 s is expected to be more automatic (Lewis and Miall, 2003). Nevertheless, it cannot be excluded that the involvement of higher cognitive functions are deployed when short intervals are processed. This involvement is expected to be task-related rather than time-related. In fact, the involvement of higher cognitive processes is expected in task that requires more cognitive control (e.g., time reproduction and time discrimination). The two studies that used short temporal intervals (between 500 and 1500 ms) reported that TBI patients were less accurate (absolute score and proportion of correct responses) than controls in particular when the standard duration was 500 ms; when relative errors were analyzed, both TBI and controls over-estimated 500 ms duration and under-estimated longer durations (1000 and 1500 ms). Consistent with previous finding obtained with longer temporal intervals, TBI patients showed higher temporal variability (Mioni et al., 2013a,b).

### Linking time perception and neuropsychological tasks

As we mentioned before, frontally mediated cognitive processes (i.e., attention, working memory, executive functions, etc.) play an important role in temporal processing (Rao et al., 2001; Perbal et al., 2002; Baudouin et al., 2006a,b). Moreover, considering that TBI patients often present frontally mediated cognitive dysfunctions, it is of interest to determine what the impact of frontally mediated cognitive impairment on time perception is. Table 2 provides a summary of correlation analyses conducted between time perception and neuropsychological tasks.

**Table 2:** **Summary table of studies that have investigated the correlation between time perception and neuropsychological tasks**.

**References**		**TBI patients**				**Controls**				**Overall**			
										**Time reproduction**		**Time production**	
										**Simple**	**Concurrent**	**Simple**	**Concurrent**
Perbal et al., 2003	**RATIO**												
	Free tempo	NA				NA				ns	ns	0.46	0.41
	1 s tempo									ns	ns	0.36	0.64
	Speed of processing									ns	ns	ns	ns
	Working memory									−0.42	ns	ns	ns
	Episodic memory									ns	ns	ns	ns
	**CV**												
	Free tempo									ns	ns	ns	ns
	1 s tempo									0.53	ns	ns	ns
	Speed of processing									−0.68	ns	0.46	ns
	Working memory									−0.50	−0.45	ns	ns
	Episodic memory									−0.50	ns	ns	ns
Schmitter-Edgecombe and Rueda, 2008		**Verbal estimation**								**Verbal estimation**			
		No significant correlations when analyses were conducted separately between TBI patients and controls *r*s = −0.38 to 0.29								Visuo-spatial memory and (a) 60 s ratio score: *r* = 0.35; (b) 25-s absolute score: *r* = −37; and (c) 45-s absolute score *r* = −0.37			
Anderson and Schmitter-Edgecombe, 2011		NA				**Verbal estimation**				NA			
						Rey Auditory Verbal Learning Test with 45 s ratio *r* = 0.60; 7/24 with 45 s ratio *r* = 0.58							
Mioni et al., 2012		**Time reproduction**				**Time reproduction**							
	**Working memory**	0.53				0.44				NA			
	**Attention**	ns				ns							
Mioni et al., 2013a		**Time Discrimination**				**Time Discrimination**							
		**500 ms**		**1300 ms**		**500 ms**		**1300 ms**		NA			
	**Working memory**	−0.49		−0.62		ns		−0.52					
	**Attention**	ns		−0.55		ns		−0.36					
	**Speed of processing**	−0.38		−0.41		ns		−0.39					
Mioni et al., 2013b										**Time reproduction**	**Time production**	**Time Discrimination**	
	**Attention**												
	Divided attention									0.46	ns	0.43	
	Go-Nogo									0.48	ns	ns	
	**Working Memory**												
	N-Back	NA				NA				0.40	ns	ns	
	Digit span backward									−0.41	ns	−0.43	
	**Executive Functions**												
	Verbal fluency									−0.51	ns	ns	
	WCST									0.60	ns	−0.54	

RATIO, relative error; CV, coefficient of variation; Simple, temporal task alone; Concurrent, temporal task + non-temporal task; NA, not available; ns, not significant.

Despite the fact that, different duration ranges are employed in different studies, and considering the fact that different studies consistently showed that different systems are involved in the processing of short (hundreds of milliseconds) and long (few seconds) temporal intervals, only three studies (Schmitter-Edgecombe and Rueda, 2008; Anderson and Schmitter-Edgecombe, 2011; Mioni et al., 2013a) reported correlation analyses between cognitive functions and different range of temporal intervals. In Mioni et al. (2013a), results showed that attention, working memory and speed of processing functions were involved when the temporal interval was 1300 ms (long standard interval) in both TBI and controls; but only in TBI patients working memory and speed of processing were involved when the standard interval was 500 ms. In the other two studies (Schmitter-Edgecombe and Rueda, 2008; Anderson and Schmitter-Edgecombe, 2011) the results showed significant correlations between longer temporal intervals (45 and 60 s) and spatial and verbal memory.

Overall, when the correlations analyses were reported, a representative index for the temporal tasks was calculated and correlated with the performance at the neuropsychological tests. Regarding the time reproduction task, significant correlations were found with the working memory index (Perbal et al., 2003; Mioni et al., 2012, 2013b^1^). Moreover, in Mioni et al. (2013b), significant correlations were also found between time reproduction index (absolute score) and attention and executive functions indices, suggesting a high involvement of cognitive resources for executing accurately the time reproduction task.

In Perbal et al. (2003), the time production index of temporal accuracy (RATIO) correlated significantly with indices of free tapping and 1-s finger tapping^2^. Moreover, the time production index of temporal variability (CV) correlated with speed of processing. In Mioni et al. (2013b), there was minimal involvement of higher order cognitive functions (attention, working memory and speed of processing) in the time production task. In both Schmitter-Edgecombe and Rueda (2008) and Anderson and Schmitter-Edgecombe (2011), significant correlations were found between verbal estimation task and indices of visuo-spatial and verbal memory tests. Finally, regarding time discrimination task, both Mioni et al. (2013a,b) reported significant correlations between time discrimination index and all measures of high cognitive functions included (attention, working memory, speed of processing, and executive functions), indicating a high involvement of cognitive resources in the time discrimination task.

### Linking time perception and clinical characteristics

Overall, the studies reported the temporal performance of 151 TBI patients (male = 86) and 129 controls (male = 79) matched by age (TBI = 35.48 years; controls = 34.10 years) and level of education (TBI = 12.01 years; controls = 12.75 years). The Glasgow Coma Scale (GCS; Teasdale and Jennett, 1974) was often used to define the severity of trauma. A score of 8 or less defines a severe TBI, a score between 9 and 12 defines moderate TBI and a score above 12 defines a mild TBI. The majority of TBI patients (115 out of 151) were scored as severe TBI, 25 were moderate TBI and 11 were mild TBI. The mean time of post-traumatic amnesia (PTA) (when available) was 33.54 days. The time between the injury and the testing varied consistently across studies from 37 days to 31.40 months. The majority of patients included where tested long time after trauma. In Meyers and Levin (1992) patients were evaluated with the Galveston Orientation and Amnesia Test (GOAT; Levin et al., 1979) and they were divided into two groups according to their orientation level. The disoriented TBI patients showed a greater under-reproduction (RATIO) of long temporal intervals (15 s) compared to controls and, in the combined TBI group, the GOAT score correlated with long interval (15 s). Schmitter-Edgecombe and Rueda (2008) and Anderson and Schmitter-Edgecombe (2011) reported the results of correlations analyses conducted between performance at the temporal tasks and injury characteristics. Surprisingly, no significant correlations were found between the verbal estimation score (RATIO) and GCS, PTA or time since injury.

## Discussion

The present work was conducted for reviewing the literature on the temporal dysfunctions of TBI patients, and for evaluating whether the temporal impairment observed is due to a disruption at the clock stage, or to the dysfunctions of the high cognitive functions involved in temporal processing. Taken together, the studies reported poorer temporal performances for TBI patients than for controls. This finding applies when investigations involve durations exceeding working memory span (Schmitter-Edgecombe and Rueda, 2008; Anderson and Schmitter-Edgecombe, 2011) or when temporal tasks require a high involvement of cognitive functions as is the case with time reproduction and time discrimination (Mioni et al., 2013a,b).

Verbal estimation and time production tasks are suitable methods to highlight variations in the internal clock rate (Block, 1990; Block et al., 1998). Lower temporal performances were observed in TBI patients when verbal estimation task was used, but only when long temporal intervals were employed (above 45 s) (Schmitter-Edgecombe and Rueda, 2008). In the case of time production, TBI were as accurate as controls both with long (4, 14, and 38 s: Perbal et al., 2003) and with short (500, 1000, and 1500 ms: Mioni et al., 2013b) intervals. The results suggest that TBI patients' temporal impairment is not due to a dysfunction at the internal clock level but to a dysfunction of high cognitive functions involved in temporal processing. This hypothesis is confirmed by the correlational analyses between time production and indices of spontaneous tempo. The positive correlation between duration production and spontaneous tempo indicated that the participants with accelerated time pacing (shorter inter-tap interval) were those who produced shorter durations, and the participants with the slower time pacing (longer inter-tap interval) were those who produced the longer durations (Perbal et al., 2003). These results are consistent with the accumulation process postulated by Church's model (1984) in which changes in the internal clock rate lead to differences in the production of the same objective target duration.

In the case of time discrimination, short temporal intervals were used to reduce the cognitive load required due to process long temporal intervals (Block et al., 2010). Significant differences were found between TBI and controls indicating that TBI were less accurate (proportion of correct responses) and more variable (CV) than controls. However, the high correlations observed between time discrimination index and high cognitive functions (i.e., attention, working memory and executive functions) suggest that lower performances observed in TBI patients are mainly due to reductions at the level of cognitive functions involved in temporal processing rather than a dysfunction at the interval clock rate (Mioni et al., 2013a,b).

More complicated are the results observed with the time reproduction task. In both Mioni et al. (2012) and Perbal et al. (2003), participants performed a time reproduction task together with a concurrent non-temporal task with durations ranging from 4 to 38 s. The authors employed a concurrent non-temporal task to prevent participants from using counting strategies (Grondin et al., 2004; Hemmes et al., 2004) and to investigate the effect of reduced attentional resources on time perception. The authors expected lower temporal performance in the concurrent (time reproduction + non-temporal task) compared to the simple (time reproduction only) condition and expected a higher effect of the non-temporal task on TBI patients due to the attentional dysfunction often observed in TBI patients (Busch et al., 2005; Boelen et al., 2009; Stuss, 2011). Both TBI and controls were less accurate in the concurrent-task condition compared to the single-task condition, confirming that time perception is influenced by attention. When attention is divided between the temporal task and the non-temporal task, less attention is dedicated to time, less pulses are accumulated and, consequently, there are under-reproductions of temporal intervals (Zakay and Block, 1996, 2004). However, the effect of non-temporal task was similar on TBI patients and controls and both groups under-reproduced temporal intervals. Different results were observed when short intervals were used (500, 1000, and 1500 ms; Mioni et al., 2013b). TBI patients were less accurate (absolute score) and more variable (CV) than controls but showed a similar pattern of under-reproduction (RATIO). It is important to note that using the time reproduction task with short intervals is highly problematic due to the motor component required to perform the task (Droit-Volet, 2010; Mioni et al., 2014). In time reproduction tasks, participants need to integrate their motor action in order to produce a precise button press to reproduce the temporal interval. Preparing and executing a motor action requires planning and execution of motor movements that might result in additional variance (Bloxham et al., 1987; Stuss et al., 1989; Caldara et al., 2004). Therefore, it is possible that the lower performances (higher absolute score and higher variability) observed were mainly due to motor dysfunctions rather than temporal impairment. In fact, neuromotor impairment is a common symptom in TBI patients, and reaction time (RT) tests with this population have consistently revealed slowness of information processing and a deficit in divided attention (Stuss et al., 1989; Walker and Pickett, 2007). Overall, the performance at time reproduction tasks is highly correlated with working memory index and with other measures of cognitive functions (i.e., attention, executive functions).

A consistent result across all studies is the higher variability observed in TBI patients compared to controls. The difficulty of maintaining a stable representation of duration might be accentuated in patients with TBI because of problems in working memory, but also in other high cognitive functions such as sustained attention or speed of processing (Brouwer et al., 1989).

Surprisingly, no strong correlations were observed between temporal performance and clinical measures. The only significant correlation was observed between the GOAT and time reproduction task at 15 s (Meyers and Levin, 1992). The GOAT includes questions about both the past and the present events and is used to help caregivers to learn when the person no longer has PTA. The significant correlation observed might explain the higher temporal variability observed in TBI patients. It is important to note that the lack of significant correlations can also be caused by the weakness of statistical power due, in most studies, to small sample sizes.

In sum, the revision of the existing literature investigating time perception in TBI patients showed that temporal dysfunctions in TBI patients were related to deficits in cognitive functions involved in temporal processing such as working memory, attention and executive functions rather than an impairment in time estimation *per se*. In fact, temporal dysfunctions were observed when the temporal intervals exceeded the working memory span (Schmitter-Edgecombe and Rueda, 2008; Anderson and Schmitter-Edgecombe, 2011) or when the tasks employed required high cognitive functions to be performed (Mioni et al., 2013a,b). The consistent higher temporal variability observed is a sign of impaired frontally mediated cognitive functions that affect temporal representation. The involvement of high cognitive functions in temporal processing is confirmed by the correlations observed between temporal tasks and working memory, attention and speed of processing in both short and long temporal intervals (Perbal et al., 2003; Schmitter-Edgecombe and Rueda, 2008; Mioni et al., 2013a,b).

## Future studies and directions

The revision of the literature investigating time perception in TBI patients showed that authors have used, over a wide range of temporal intervals (from 500 ms to 60 s) and the classical time perception methods (Grondin, 2008, 2010). Despite the limited number of studies, the results point in the same direction and show that temporal dysfunction in TBI patients is mainly a secondary impairment due to deficits in the cognitive functions involved in temporal processing rather than to an impairment in time estimation *per se*. However, more studies should be conducted for drawing a more complete picture of the temporal dysfunctions in TBI patients, or of the source of these dysfunctions.

Future studies should assess the temporal performances in tasks where time is marked by stimuli delivered from different modalities. All the studies conducted used visual stimuli, and it is well-known that the nature of the stimuli (i.e., visual, auditory, tactile) influences temporal performance (Grondin, 2010). In particular, temporal sensitivity is higher when the stimuli are presented in the auditory modality rather than in the visual modality (Grondin, 1993; Grondin et al., 1998). By reducing the noise produced by the presentation of visual stimuli marking time, chances are probably increased to access the sources of temporal variability in TBI performances and to disentangle the variability produced by clinical characteristics and the variability due to some methodological characteristics.

Moreover, future studies should investigate the effects of emotion on time perception in TBI patients. The literature reveals that marking time with images of faces expressing different emotions can affect time perception. Facial expressions of anger, fear, happiness, and sadness generate an overestimation of time, but the facial expression of shame generates an underestimation of time (Gil and Droit-Volet, 2011a,b). Some studies also have shown that the ability to read emotion in other people's faces can be selectively impaired as a result of the head injury (Jackson and Moffat, 1987; Bornstein et al., 1989; Fleming et al., 1996; Green et al., 2004; Martins et al., 2011). Investigating the effect of emotion on time perception in TBI patients can provide important information regarding the degree of emotional impairment in TBI patients.

Finally, some studies have shown that time perception (as measured in time estimation and time production tasks) may be related to impulsiveness (Barratt and Patton, 1983; Stanford and Barratt, 1996). In particular, the internal clocks of impulsive individuals may run faster than those of non-impulsive individuals (Barratt and Patton, 1983); therefore, an impulsive individual would likely experience some temporal distortions (Van denBroek et al., 1992). TBI patients often demonstrate impulsive behavior, in particular after damage to the orbitofrontal cortex (Berlin et al., 2004). Although, there is no clear evidence of a specific contribution of orbitofrontal cortex on time perception vs. other parts of frontal cortex, it is of interest to further investigate the different contribution of frontal areas on time perception and distinguish how impulsivity, personality, and cognitive dysfunctions are involved in the temporal dysfunctions.

### Conflict of interest statement

The authors declare that the research was conducted in the absence of any commercial or financial relationships that could be construed as a potential conflict of interest.

## Footnotes

^1^Meyers and Levin (1992) is the fourth study that used a time reproduction task but no correlations with neuropsychological tasks are included.

^2^In the finger-tapping task, participants were required to tap with their index finger, as regularly as possible at the pace they preferred (free tempo) or at a 1 s pace (1 s tempo) (Perbal et al., 2003).
